# Automatic Sleep Stage Scoring Using Time-Frequency Analysis and Stacked Sparse Autoencoders

**DOI:** 10.1007/s10439-015-1444-y

**Published:** 2015-10-13

**Authors:** Orestis Tsinalis, Paul M. Matthews, Yike Guo

**Affiliations:** Department of Computing, Imperial College London, London, UK; Division of Brain Sciences, Imperial College London, London, UK

**Keywords:** Electroencephalography, EEG, Deep learning, Ensemble learning

## Abstract

We developed a machine learning methodology for automatic sleep stage scoring. Our time-frequency analysis-based feature extraction is fine-tuned to capture sleep stage-specific signal features as described in the American Academy of Sleep Medicine manual that the human experts follow. We used ensemble learning with an ensemble of stacked sparse autoencoders for classifying the sleep stages. We used class-balanced random sampling across sleep stages for each model in the ensemble to avoid skewed performance in favor of the most represented sleep stages, and addressed the problem of misclassification errors due to class imbalance while significantly improving worst-stage classification. We used an openly available dataset from 20 healthy young adults for evaluation. We used a single channel of EEG from this dataset, which makes our method a suitable candidate for longitudinal monitoring using wearable EEG in real-world settings. Our method has both high overall accuracy (78%, range 75–80%), and high mean $F_1$-score (84%, range 82–86%) and mean accuracy across individual sleep stages (86%, range 84–88%) over all subjects. The performance of our method appears to be uncorrelated with the sleep efficiency and percentage of transitional epochs in each recording.

## Introduction

Sleep is central to human health. The health consequences of reduced sleep, abnormal sleep patterns or desynchronized circadian rhythms can be emotional, cognitive, or somatic.^26^ Associations between disruption of normal sleep patterns and neurodegenerative diseases are well recognized.^26^

According to the American Academy of Sleep Medicine (AASM) manual,^11^ sleep is categorized into four stages. These are rapid eye movement (stage R) sleep and 3 non-R stages, stages N1, N2 and N3. Formerly, stage N3 (also called slow wave sleep, or SWS) was divided into two distinct stages, N3 and N4.^20^ To these a wake (W) stage is added. These stages are defined by electrical activity recorded from sensors placed at different parts of the body. The totality of the signals that are recorded through these sensors is called a polysomnogram (PSG). The PSG includes an electroencephalogram (EEG), an electrooculogram (EOG), an electromyogram (EMG), and an electrocardiogram (ECG). After the PSG is recorded, it is divided into 30-s intervals, called *epochs*. Then, one or more experts classify each epoch into one of the five stages (N1, N2, N3, R or W) by quantitatively and qualitatively examining the signals of the PSG in the time and frequency domains. Sleep scoring is performed according to the Rechtschaffen and Kales sleep staging criteria.^20^ In Table 1 we reproduce the Rechtschaffen and Kales sleep staging criteria,^22^ merging the criteria for N3 and N4 into a single stage (N3).

**Table 1:** The Rechtschaffen and Kales sleep staging criteria,^20^ adapted from.^22^

Sleep stage	Scoring criteria
Non-REM 1 (N1)	50% of the epoch consists of relatively low voltage mixed (2–7 Hz) activity, and <50% of the epoch contains alpha (8–13 Hz) activity. Slow rolling eye movements lasting several seconds often seen in early N1.
Non-REM 2 (N2)	Appearance of sleep spindles and/or K-complexes and <20% of the epoch may contain high voltage (>72 *μ*V, <2 Hz) activity. Sleep spindles and K-complexes each must last >0.5 s.
Non-REM 3 (N3)	20–50% (formerly N3) or >50% (formerly N4) of the epoch consists of high voltage (>75 *μ*V), low frequency (<2 Hz) activity.
REM (R)	Relatively low voltage mixed (2–7 Hz) frequency EEG with episodic rapid eye movements and absent or reduced chin EMG activity.
Wake (W)	>50% of the epoch consists of alpha (8–13 Hz) activity or low voltage, mixed (2–7 Hz) frequency activity.

Recent research suggests that detection of sleep/circadian disruption could be a valuable marker of vulnerability and risk in the early stages of neurodegenerative diseases, such as Alzheimer’s disease, Parkinson’s disease and multiple sclerosis, and that sleep stabilization could improve the patients’ quality of life.^26^ There is therefore a pressing need for longitudinal sleep monitoring for both medical research and medical practice. In this case an affordable, portable and unobtrusive sleep monitoring system for unsupervised at-home use would be ideal. Wearable EEG is a strong candidate for such use. A core software component of such a system is a sleep scoring algorithm, which can reliably perform automatic sleep stage scoring given the patient’s EEG signals.

In this study we present and evaluate a machine learning methodology for automatic sleep stage scoring using a single channel of EEG. Our methodology is based on time-frequency analysis^4^ and stacked sparse autoencoders (SSAEs).^1^ We compared the performance of our method with three existing studies. In^6^ the data consisted of 16 subjects (aged 30–75 years) and the EEG channel used was C3-A1. The authors’ method was time-frequency analysis using the continuous wavelet transform (CWT) and Renyi’s entropy for feature extraction, and the random forest classifier. In^16^ the first dataset comprised 20 subjects (aged 20–22 years), using channel C3-A2. The second dataset^19^ comprised eight subjects (aged 21–35 years), and the authors chose channel Pz-Oz. The authors used multiscale entropy (MSE) for feature extraction from the EEG signal, and they also fitted an autoregressive (AR) model to the signal. They then trained a linear discriminant analysis (LDA) model using the MSE features and the fitted parameters of the AR model as features, employing a set of 11 *a priori* ‘smoothing rules’ on the hypnogram after the initial sleep scoring. In^3^ the authors used a dataset comprising 15 subjects (aged 29.2 $\pm$ 8 years). The feature extraction methods and the machine learning algorithm are not described in detail in Ref. 3.

There are two main limitations in the existing literature. First, regarding the results of the proposed methods, in all three studies we observe imbalance in the scoring performance across sleep stages. For example, the $F_1$-score in the worst-classified sleep stage (N1) can be as low as 30% in Ref. 16. Second, regarding the evaluation methodology, in all three studies the authors evaluated their methods using a single training-testing split of the data, and did not perform any type of cross-validation. Furthermore, in Ref. 6 the authors trained and tested their algorithm using epochs from all subjects, which means that the training and testing datasets were not independent. In this work we mitigated skewed sleep scoring performance in favor of the most represented sleep stages, and addressed the problem of misclassification errors due to class imbalance in the training data while significantly improving worst-stage classification. Our experimental design employs cross-validation across subjects, ensuring independence of training and testing data.

## Materials and Methods

### Data

The dataset that we used to evaluate our method is a publicly available sleep PSG dataset^14^ from the PhysioNet repository^7^ that can be downloaded from.^18^ The data was collected from electrodes Fpz-Cz and Pz-Oz, instead of the standard C3-A2 and C4-A1. The sleep stages were scored according to the Rechtschaffen and Kales guidelines.^20^ The epochs of each recording were scored by a single expert (6 experts in total). The sleep stages that are scored in this dataset are wake (W), REM (R), non-R stages 1–4 (N1, N2, N3, N4), movement and not scored. For our study, we removed the very small number of movement and not scored epochs (not scored epochs were at the start or end of each recording), and also merged the N3 and N4 stages into a single N3 stage, as it is currently the recommended by the AASM.^11^,^22^ There were 61 movement epochs in our data in total, and only 17 of the 39 recordings had movement artifacts. The maximum number of movement epochs per recording was 12. The rationale behind the decision of removing the movement epochs was based on two facts. First, these epochs had not been scored by the human expert as belonging to any of the five sleep stages, as it is recommended in the current AASM manual.^11^^, p. 31^ Second, their number was so small that they could not be used as a separate ‘movement class’ for learning. The public dataset includes 20 healthy subjects, 10 male and 10 female, aged 25–34 years. There are two approximately 20-h recordings per subject, apart from a single subject for whom there is only a single recording. To evaluate our method we used the in-bed part of the recording. The sampling rate is 100 Hz and the epoch duration is 30 s.

### Feature Extraction

For feature extraction we performed time-frequency analysis using complex Morlet wavelets (see, for example, Chapters 12 and 13, pp. 141–174 in Ref. 4). The reason for preferring a time-frequency-based feature extraction method over the Fourier transform was that we wanted to extract features that capture the mixture of frequencies and their interrelations at different points in time as features.

For time-frequency analysis using complex Morlet wavelets there are two sets of parameters that need to be chosen, the peak frequencies and the number of wavelet cycles per frequency. The number of wavelet cycles defines its width and controls the trade-off between temporal and frequency precision. Specifically, increasing the number of cycles increases the frequency precision but decreases the temporal precision, while decreasing the number of cycles increases the temporal precision but decreases the frequency precision. In this study we selected the peak frequencies and the number of cycles based on the sleep scoring criteria in Table 1, taking into account the transition rules in Table 2. In Table 3 we summarize the parameters chosen.

**Table 2:** The transition rules summarized from the AASM sleep scoring manual.^11^ ^Chapter IV: Visual Rules for Adults, pp. 23–31]^

Stage pair	Transition pattern	Rule	Differentiating features
N1–N2	N1-{N1,N2}	5.A.Note.1	Arousal, K-complexes, sleep spindles
	(N2-)N2-{N1,N2}(-N2)	5.B.1	K-complexes, sleep spindles
		5.C.1.b	Arousal, K-complexes, sleep spindles
	N2-{N1-N1,N2-N2}-N2	5.C.1.c	Alpha, body movement, slow eye movement
N1-R	R-R-{N1,R}-N2	7.B	Chin EMG tone
		7.C.1.b	Chin EMG tone
		7.C.1.c	Chin EMG tone, arousal, slow eye movement
	R-{N1-N1-N1,R-R-R}	7.C.1.d	Alpha, body movement, slow eye movement
N2-R	R-R-{N2,R}-N2	7.C.1.e	Sleep spindles
	(N2-)N2-{N2,R}-R(-R)	7.D.1	Chin EMG tone
		7.D.2	Chin EMG tone, K-complexes, sleep spindles
		7.D.3	K-complexes, sleep spindles

Curly braces indicate choice between the stages or stage progressions in the set based on the distinctive features, and parentheses indicate optional epochs

**Table 3:** Peak frequencies and number of wavelet cycles per frequency for time-frequency analysis using complex Morlet wavelets.

Target frequency band	Target sleep stages	Frequency or time precision	Peak frequency (Hz)	Number of wavelet cycles
Slow (0.5–2 Hz)	N3	Time	0.7	3
Slow (0.5–0 Hz)	N3	Time	1	3
Slow (0.5–2 Hz)	N3	Time	1.5	3
Slow (0.5–2 Hz)	N3	Time	2	3
K-complex (1.6–4 Hz)^9^	N2	Time	2	3
K-complex (1.6–4 Hz)^9^	N2	Time	3.2	3
delta/theta (2–7 Hz)	N1,R,W	Intermediate	3	5
delta/theta (2–7 Hz)	N1,R,W	Intermediate	4	5
delta/theta (2–7 Hz)	N1,R,W	Intermediate	5	5
delta/theta (2–7 Hz)	N1,R,W	Intermediate	6	5
alpha (8–13 Hz)	N1,W	Frequency	8	10
alpha (8–13 Hz)	N1,W	Frequency	10	10
alpha (8–13 Hz)	N1,W	Frequency	12	10
Spindle (12–15 Hz)	N2,N3	Time	12	3
Spindle (12–15 Hz)	N2, N3	Time	13	3
Spindle (12–15 Hz)	N2,N3	Time	14	3
Spindle (12–15 Hz)	N2,N3	Time	15	3
beta (15–30 Hz)	N1 (arousal)	Time	16	3
beta (15–30 Hz)	N1 (arousal)	Time	18	3
beta (15–30 Hz)	W	Intermediate	20	5
gamma (30–100 Hz)^a^	N1,N2,N3,R,W	Intermediate	40	5

^a^ There is evidence in the literature that features from modalities other than EEG, such as eye movements,^27^ stage R sleep^13^ and EMG activity,^8^,^25^ can manifest themselves in the gamma activity of EEG

After extracting the frequency-band power for each peak frequency given in Table 3, the features that we computed for each epoch were the power of the frequency-band power signal, the power of the time-domain signal, the Pearson correlation coefficient between each pair of frequency-band power signals and the autocorrelation in the time-domain signal for 50 time lags (i.e., up to 0.5 s). Additionally, we used a sliding window to extract the power of the frequency-band power and the power of the time-domain signal at different intervals within each epoch. Specifically, we used a sliding window of duration of 5 s and step of 2.5 s, which resulted in 11 power of frequency-band power features per frequency band per epoch and 11 power of the time-domain signal features per epoch. All the extracted features are summarized in Table 4. We mapped all the features in the [0,1] interval, and centered their distribution using transformations (see Table 4), as this is beneficial for our learning algorithm. We then normalized the features from each trial of each subject.

**Table 4:** Features extracted from the single channel EEG signal.

Feature	Number	Purpose	Transform
Power of frequency-band power over the entire epoch	22	Capture the overall presence of the particular frequency band in the signal	$\log (x)$
Power of frequency-band power using a sliding window	231	Capture the presence of the particular frequency band in the signal across time	$\log (x)$
Time-domain signal power over the entire epoch	1	Capture the overall amplitude characteristics of the signal	$\log (x)$
Time-domain signal power using a sliding window	11	Capture the amplitude characteristics of the signal over time	$\log (x)$
Frequency-band power-power correlation	242	Capture the relationships between the different frequency bands over time	None
Time-domain signal autocorrelation	50	Capture long-term dependencies in the signal	$x^2$
ALL	557		

The AASM manual^11^ includes a number of rules that recommend taking into account neighboring epochs for the scoring of each current epoch under certain circumstances. We identified 12 rules in total concerning the transition between certain sleep stage pairs that refer to seven distinct transition patterns, as shown in Table 2. These rules apply to three sleep stage pairs, N1–N2, N1-R and N2-R. The transition patterns include up to two preceding or succeeding neighboring epochs. Trying to capture the effect of these transition rules in an automatic sleep scoring algorithm by simply including transition probabilities between sleep stages is not a suitable approach. The reason is that the algorithm could overfit to hypnogram-level patterns from the subjects we used for training, especially when the training data do not include data from different sleep pathologies.

We incorporated transition information directly as features for our machine learning algorithm. Specifically, for the classification of each epoch, apart from the features corresponding to itself, we included the features from the preceding two and succeeding two epochs. We addressed the possibility of overfitting which exists in this case in our experimental design ("Evaluation" Section). In the literature, Liang *et al.*^16^ used 11 *a priori* hypnogram ‘smoothing rules’ in order to capture transition information. These rules are applied on the scored epochs after automatic sleep scoring has taken place, effectively changing the classification of each epoch given the sleep stage of its neighbors. Unfortunately, the authors described only two of the rules in their paper, and, notably, did not discuss the order in which the rules are applied to the estimated hypnogram.

### Machine Learning Methodology

Stacked sparse autoencoders^1^ are a specific type of neural network model. The key difference between stacked autoencoders and standard neural networks is layer-wise pre-training using unlabelled data (i.e., without class labels) before fine-tuning the network as a whole.^2^ Autoencoders are trained using iterative optimisation with the backpropagation algorithm. The optimisation method we used was L-BFGS, as recommended in Ref. 15. The hyperparameters of a sparse autoencoder-based model are: (1) a regularization weight $\lambda$ which is used to decrease the magnitude of the parameters and prevent overfitting, (2) a sparsity weight $\beta$ which controls the relative importance of the sparsity penalty term, (3) a sparsity parameter $\rho$ which sets the desired level of sparsity, and (4) the number of units *n* in the hidden layer of the autoencoder. The only hyperparameter for the optimisation is the total number of iterations *r*.

The combinatorial space to explore all the possible combinations of hyperparameters is huge. Therefore, we decided to choose the same hyperparameters across all layers. Our final choice was $\lambda =1 \times 10^{-5}$, $\beta =2.0$, $\rho =0.2$, $n=20$, and $r=60$. We used autoencoders with the sigmoid activation function, which is symmetric. This is the reason that our features were transformed so that their distribution be approximately centered around the mean.

The classes (sleep stages) in our dataset, as in any PSG dataset, were not balanced, i.e., there were a lot more epochs for some stages (particularly N2) than others (particularly W and N1). In such a situation, if all the data is used as is, it is highly likely that a classifier will exhibit skewed performance favoring the most represented classes, unless the least represented classes are very distinct from the other classes. In order to resolve the issues stemming from imbalanced classes we decided to employ class-balanced random sampling with an ensemble of classifiers, each one being trained on a different sample of the data. Our final model consisted of an ensemble of 20 independent SSAEs with the same hyperparameters. Each of the 20 SSAEs was trained using a sample of the data in which the number of epochs per-stage per recording was equal to the number of epochs of the least represented stage (N1). The classification of the epochs in the testing recordings was done by taking the mean of the class probabilities that each of the 20 SSAEs outputs, and then selecting the class with the highest probability.

We used our own Matlab implementation for time-frequency analysis and stacked autoencoders, and the Matlab implementation by Mark Schmidt for L-BFGS (http://www.cs.ubc.ca/~schmidtm/Software/minFunc.html).

### Evaluation

To evaluate the generalisability of our method, we obtained our results using 20-fold cross-validation. Specifically, in each fold we used the recordings of a single subject for testing and all other recordings for training. We used each subject’s recordings only once for testing, thus obtaining a one-to-one correspondence of cross-validation folds and test subjects. We chose per subject cross-validation as we also performed comparisons across individual recordings. With this experimental design, we were able to assess both the overall performance of our method and the performance across recordings with a single set of experimental results.

We report the evaluation metrics using their average across all recordings. Specifically, we report their mean value across all five sleep stages and their value for the most misclassified sleep stage, which gives information about the robustness of the method across sleep stages. We tested our method with both available EEG electrodes (Fpz-Cz and Pz-Oz). We report the scoring performance using the best electrode, which was Fpz-Cz. Finally, we calculated 95% confidence intervals for each of the performance metrics by bootstrapping using 1000 bootstrap samples across the 39 recordings.

We also tested our algorithm using fivefold cross-validation with non-independent training and testing sets by mixing the subjects’ epochs as the authors in Ref. 6 did. This was done to show the improvement in the results that such a flawed practice can result into, and appropriately compare our method to.^6^ We do not consider this performance indicative of the quality of our method, or any method targeted in EEG sleep scoring, as it is not practical in the real world. These results are separated from the others in Table 6.

To further evaluate the generalisability of our method, we performed two tests on our results to assess the correlation between scoring performance and (1) a measure of the sleep quality of each recording, and (2) the percentage of transitional epochs in each recording. Robust scoring performance across sleep quality and temporal sleep variability, can be seen as further indicators of the generalisability of an automatic sleep stage scoring algorithm. The reason is that low sleep quality and high sleep stage variability across the hypnogram are prevalent in sleep pathologies (see, for example^17^).

We measured sleep quality with a widely-used index, called *sleep efficiency*. Sleep efficiency is defined as the percentage of the total time in bed that a subject was asleep.^23^^, p.226^ Our data contain a ‘lights out’ indicator, which signifies the start of the time in bed. We identified the sleep onset as the first non-W epoch that occurred after lights were out. We identified the end of sleep as the last non-W epoch after sleep onset, as our dataset does not contain a ‘lights on’ indicator. The number of epochs between the start of time in bed and the end of sleep was the total time in bed, within which we counted the non-W epochs; this was the total time asleep. We defined transitional epochs as those whose preceding or succeeding epochs were of a different sleep stage than them. We computed their percentage with respect to the total time in bed. In our experiments we computed the $R^2$ and its associated* p*-value between sleep efficiency and scoring performance, and between percentage of transitional epochs and scoring performance.

All scoring performance metrics are derived from the confusion matrix. Using a ‘raw’ confusion matrix in the presence of imbalanced classes implicitly assumes that the relative importance of correctly detecting a class is directly proportional to its frequency of occurrence. This is not desirable for sleep staging. What we need to mitigate the negative effects of imbalanced classes on classification performance measurement is effectively a normalized or ‘class-balanced’ confusion matrix that places equal weight into each class. Surprisingly, in the single channel EEG sleep staging literature there are examples of such mistakenly reported performance results using the raw confusion matrix. For this reason, we compared our work only with the studies in the literature that provided the raw confusion matrix, from which we computed the performance metrics after class-balancing.

The metrics we computed were precision, sensitivity, $F_1$-score, per-stage accuracy, and overall accuracy. The $F_1$-score is the harmonic mean of precision and sensitivity and is a more comprehensive performance measure than precision and sensitivity by themselves. The reason is that precision and sensitivity can each be improved at the expense of the other. All the metrics apart from overall accuracy are binary. However, in our case we have five classes. Therefore, after we performed the classification and computed the normalized confusion matrix, we converted our problem into five binary classification problems each time considering a single class as the ‘positive’ class and all other classes combined as a single ‘negative’ class (*one-*vs.*-all* classification).

Finally, we computed the scoring performance of our algorithm without and with features from neighboring epochs. If we observed improvement in sleep stage pairs which are not included in the transition rules (i.e., any pair other than N1–N2, N1-R and N2-R, see Table 2), we would conclude that the algorithm learned spurious patterns that are an artifact of our training data. Additionally, we should observe at least some small improvement and certainly no decrease in the classification performance between pairs N1–N2, N1-R and N2-R. In this case, even without having data from different sleep pathologies we can evaluate whether the epoch-to-epoch or hypnogram-level patterns that our algorithm learned were akin to the generic guidelines or overfitting to the training data.

## Results

As we show in the the normalized confusion matrix in Table 5, the most correctly classified sleep stage was N3, with around 90% of stage N3 epochs correctly classified. Stages N2, R and W follow, with around 80% of epochs correctly classified for each stage. The most misclassified stage was N1 with 60% of stage N1 epochs correctly classified. Most misclassifications occurred between the pairs N1-W and N1-R (about 15 and 13% respectively), followed by pairs N1–N2 and N2–N3 (about 8%), and N2-R and R-W (about 4%). The remaining pairs had either misclassification rates smaller than 4% (N2-W and N3-W) or almost no misclassifications at all (N1–N3 and N3-R). We also observe that the percentage of false negatives with respect to each stage (non-diagonal elements in each row) per pair of stages was approximately balanced between the stages in the pair (the only conspicuous exception is the pair N1-W, and, to a lesser extent, the pair N2-W). Effectively the upper and lower triangle of the confusion matrix are close to being mirror images of each other. This is a strong indication that the misclassification errors due to class imbalance have been mitigated.

**Table 5:** Confusion matrix from cross-validation using the Fpz-Cz electrode.

	N1 (algorithm)	N2 (algorithm)	N3 (algorithm)	R (algorithm)	W (algorithm)
N1 (expert)	**1654** (60%)	**262** (9%)	**8** (0%)	**366** (13%)	**472** (17%)
N2 (expert)	**1270** (7%)	**13,696** (78%)	**1231** (7%)	**760** (4%)	**621** (4%)
N3 (expert)	**7** (0%)	**469** (8%)	**4966** (89%)	**6** (0%)	**143** (3%)
R (expert)	**899** (12%)	**340** (4%)	**0** (0%)	**6164** (80%)	**308** (4%)
W (expert)	**441** (13%)	**34** (1%)	**23** (1%)	**138** (4%)	**2744** (81%)

This confusion matrix is the sum of the confusion matrices from each fold. The numbers in bold are numbers of epochs. The numbers in parentheses are the percentage of epochs that belong to the class classified by the expert (rows) that were classified by our algorithm as belonging to the class indicated by the columns

As we show in Table 6, our method has both high overall accuracy (78%, range 75–80%), and high mean F1-score (84%, range 82–86%) and mean accuracy across individual sleep stages (86%, range 84–88%) over all subjects. From the scoring performance metrics results in Table 6 we observe that our method either outperformed or had approximately equal performance with the methods in the literature in all metrics apart from worst-stage precision (the non-independent testing results at the bottom row are not taken into account). In many cases, even the lower end of the 95% confidence interval (the top number in parentheses) was higher than the corresponding metric for the other methods. Table 6 also summarizes the improvement of our method over the state of the art, i.e., the best of all the methods in the literature in that particular metric (negative numbers indicate worse performance than the state of the art). Overall, our method exhibits improved performance over the state of the art in automated sleep scoring using single channel EEG across the five scoring performance metrics.

In Table 7 we show the results of the algorithm without and with information from neighboring epochs. We observe that there is no mutual improvement in any other stages apart from the targeted pairs N1–N2, N1-R and N2-R.

**Figure 1:**
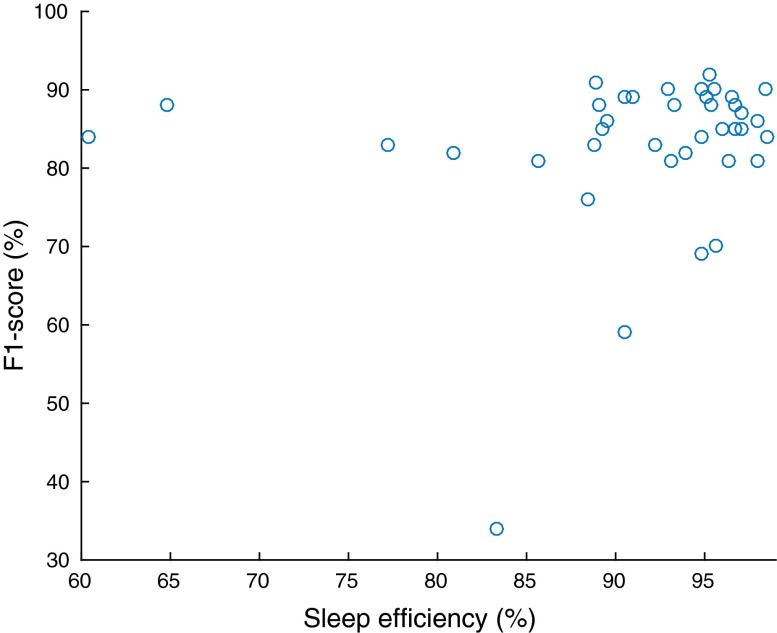
$F_1$-score as a function of sleep efficiency.

**Figure 2:**
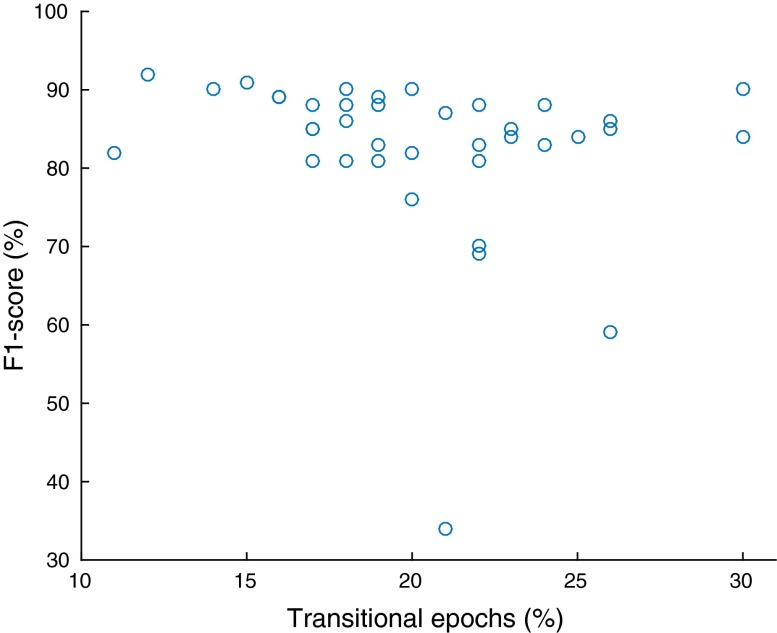
$F_1$-score as a function of transitional epochs.

We also assessed the independence of the scoring performance (for $F_1$-score and overall accuracy) of our method across recordings relative to sleep efficiency and the percentage of transitional epochs per recording (Table 8). The p-values of the regression coefficients are all above 0.15, which means that we fail to reject the null hypothesis of zero $R^2$, which is already negligible (lower than 0.1) in all cases. For clarity we present the data for these tests graphically for the $F_1$-score results in Figs. 1 and 2. Our dataset contained 10 recordings with sleep efficiency below 90% (in the range 60–89%), which is the threshold recommended in Ref. 23, p. 7 for young adults. The percentage of transitional epochs ranged from 10–30% across recordings.

**Figure 3:**
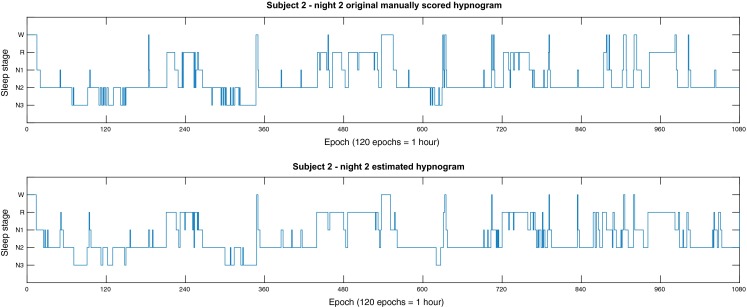
The original manually scored hypnogram (top) and the estimated hypnogram using our algorithm (bottom) for the second night of subject number 2.

Finally, in Fig. 3 we present an original manually scored hypnogram and its corresponding estimated sleep hypnogram using our algorithm for a single PSG for which the overall $F_1$-score was approximately equal to the mean $F_1$-score across the entire dataset.

**Table 6:** Comparison between our method and the literature across the five scoring performance metrics (precision, sensitivity, $F_1$-score, per-stage accuracy, and overall accuracy).

	Scoring performance metrics								
	Precision		Sensitivity		$F_1$-score		Accuracy		
**Study**	Mean	Worst	Mean	Worst	Mean	Worst	Mean	Worst	Overall
Independent training and testing									
Ref. 16	93	**89**	77	29	82	43	86	63	77
Ref. 16	90	82	73	19	77	31	83	57	73
Ref. 3	92	88	74	36	81	51	84	66	74
	(92)	(86)	(75)	(55)	(82)	(68)	(84)	(74)	(75)
Current	**93**	88	**78**	**60**	**84**	**71**	**86**	**76**	**78**
	(94)	(90)	(80)	(65)	(86)	(75)	(88)	(78)	(80)
	*0*	*−1*	*+1*	*+24*	*+2*	*+20*	*0*	*+10*	*+1*
Non-independent training and testing									
Ref. 6	93	88	77	53	84	68	86	75	77
Current	**95**	**91**	**82**	**65**	**88**	**76**	**89**	**79**	**82**
	*+2*	*+3*	*+5*	*+8*	*+4*	*+8*	*+3*	*+4*	*+5*

For the binary metrics, we report the mean performance (over all five sleep stages) as well as the worst performance (in the most misclassified sleep stage, always stage N1). We present the results for our method using the Fpz-Cz electrode with cross-validation using both independent and non-independent training and testing. The numbers in parentheses are the bootstrap 95% confidence interval bounds for the mean performance across subjects. The signed numbers in italics indicate the improvement (positive) or deterioration (negative) in performance over the second best (improvement) or best (deterioration) method in the literature

**Table 7:** Normalized confusion matrices from 20-fold cross-validation using the Fpz-Cz electrode without and with neighboring epochs. All values are percentages. Pairs of stages with mutual improvement are in bold (N1–N2, N1-R and N2-R).

	Algorithm									
	Without neighboring epochs					With neighboring epochs				
	N1	N2	N3	R	W	N1	N2	N3	R	W
N1 (expert)	53	**11**	0	**17**	18	60	**9**	0	**13**	17
N2 (expert)	**8**	77	7	**5**	4	**7**	78	7	**4**	4
N3 (expert)	0	8	89	0	3	0	8	89	0	3
R (expert)	**18**	**5**	0	73	5	**12**	**4**	0	80	4
W (expert)	13	1	1	4	82	13	1	1	4	81

**Table 8:** Correlation between sleep efficiency and percentage of transitional epochs, and scoring performance ($F_1$-score and overall accuracy).

Metric	Recording parameters			
	Sleep efficiency		Percentage of transitional epochs	
	$R^2$	*p*-value	$R^2$	*p*-value
$F_1$-score	0.02	0.42	0.04	0.20
Overall accuracy	0.02	0.46	0.05	0.17

## Discussion

Given the high disagreement across epochs between human experts^24^ a 1–2% improvement in mean scoring performance may not be considered significant. We think that there are two characteristics that render our method better than the state of the art. First, we significantly decreased the gap between the mean performance over all sleep stages and the most misclassified stage performance (stage N1) compared to the state of the art with about 20% improvement in the $F_1$-score and 10% improvement in accuracy over the state of the art (with independent testing). Second, we mitigated the adverse effects of class imbalance to sleep stage scoring. This is an indication that our method could be generalized to data with varying proportions across sleep stages, and is not markedly affected by these proportions, as other methods in the literature seem to be by inspecting their normalized confusion matrices. In our future work we aim to replicate these results in independent datasets. After addressing class imbalance, the majority of the remaining misclassification errors is likely due to either differences in EEG patterns that our feature extraction methodology cannot sufficiently capture, difficulty in capturing EOG and EMG-related that are important in distinguishing between certain sleep stage pairs features through the single channel of EEG, or inherent similarities between sleep stages in epochs that even experts would disagree with one another about.

The most misclassified pair of sleep stages using our method was N1-W; about 15% false negatives for each stage were accounted for by the other. We think that the root cause of the problem is the similarity in the characteristic EEG frequency patterns of sleep stages N1 and W, as described in the AASM sleep scoring manual.^11^ Specifically, relatively low voltage mixed 2–7 Hz and alpha (8–13 Hz) activity are described as criteria for both stages. The second most misclassified pair of sleep stages was N1-R, for which the characteristic EEG frequency patterns are similar as well. There are four transition rules which pertain to the N1-R pair in the AASM manual, which have proven useful, as we showed in Table 7. However, some of these rules rely heavily on EOG and EMG, so it was difficult to exploit their full potential. The next most misclassified pairs of sleep stages were N1–N2 and N2–N3 (about 8%). The classification between stages N1 and N2 depends to a great extent on transition patterns (Table 2) that partly rely on the detection of arousals (and, in particular, on K-complexes associated or not with arousals), body movements and slow eye movements, which can be difficult to capture using a single channel of EEG. The misclassification between stages N2 and N3 could be partly attributed to the potential persistence of sleep spindles in stage N3.^11^^, p. 27^

Of the two electrodes in the dataset, we achieved better results using the signal from electrode Fpz-Cz. We hypothesized that this was due to fact that the Fpz-Cz position can better capture most of the frequency band activity that is important for sleep staging. Specifically, delta activity.^5^ K-complexes^9^ and lower frequency sleep spindles^12^ are predominantly frontal phenomena, and alpha activity, although it is predominantly an occipital phenomenon, can manifest itself in frontal derivations.^5^. Theta activity^5^ and higher frequency sleep spindles^12^ are mostly parietal phenomena. However, theta activity is present in multiple sleep stages, so even if it were captured more effectively from the Pz-Oz position it might not have been very beneficial by itself. In our future work, we aim to work with datasets with more electrodes so that we can rigorously test specific hypotheses about the suitability of different electrode positions.

Although we recognize that our dataset does not contain a very large number of recordings of bad sleep quality, we found no statistically significant correlation between sleep efficiency and mean scoring performance. Similarly, there was no statistically significant correlation between the percentage of transitional epochs (which are by definition more ambiguous) and mean sleep scoring performance. These statistical test results indicate that our method could be robust across a number of potentially adverse factors. In our future work we aim to perform the same tests in datasets containing a wider range of ages and sleep pathologies.

Mean interrater agreement between human sleep scorers across subjects and stages can vary significantly. For example, in Ref. 24 the consensus agreement among three experts was between 60 and 80%. It would therefore be desirable that the difference in the performance of an automated scoring algorithm across scorers is not significant (i.e., that the algorithm does not overfit to a specific expert’s scoring style). Each recording in our dataset was scored by one of six different experts. In total there are 27 recordings scored by a single expert (expert C), and 12 recordings scored by all other five experts combined. The number of recordings per expert was not sufficiently large to perform a formal statistical test to assess the significance of differences in scoring performance across experts. Both the mean $F_1$-score for the recordings scored by expert C and the mean $F_1$-score for the recordings scored by any of the other experts were between 83–84%. Both values are close to each other and the overall $F_1$-score. In our future work we aim to work with datasets that either, preferably, are scored using consensus agreement or, alternatively, contain a larger number of recordings per expert.

For different pathologies that are related with sleep disorders, there are different sleep stages that are relatively more important for distinguishing them from normal sleep. For instance, to distinguish normal sleep from sleep in patients with depression stages R and N3 are relatively more important than other stages (see for example^21^). Common measures of sleep quality, include sleep efficiency, wake after sleep onset and sleep latency,^23^^, p. 226^ for all of which detection of stage W is essential. Different drugs are associated with effects in all non-R sleep stages N1, N2 and N3.^23^^, p. 9^ Excessive daytime sleepiness and sudden-onset sleep (sudden W to N2 transition) are present in Parkinson’s disease,^10^ and detection of stages N1 and N2 are particularly important for those. These examples indicate the broad range of sleep architecture aspects that need to be targeted across different pathologies. Therefore, the accurate scoring of the entire sleep architecture would be beneficial for a wide range of biomedical applications.

Our method can account for case-specific relative importance of sleep stages in a straightforward way. Our classification algorithm outputs class probabilities. Since in our paper we placed the same weight to each sleep stage, we classified each epoch to the stage that had the highest class probability. If we wanted to place different weight to each class, we could multiply each stage’s probability with a stage-specific weight before choosing the stage with the highest class probability (of course, these weights should be the same for each classified epoch). This would incorporate the relative importance that a researcher places on each sleep stage given the specific sleep pathology that they are trying to identify.

To the best of our knowledge our method has the best performance in the literature when classification is done across all five sleep stages simultaneously using a single channel of EEG. This is different from doing fewer than five one-vs.-all classification tasks, as in the latter case, if the eventual overall objective is simultaneous 5-class classification, the performance is likely overestimated. There are examples in the literature that achieve higher performance in a single or two one-vs.-all classification tasks, especially for the most easily distinguishable stages N3 and W. However, this is not the same as achieving high performance in a 5-class classification problem, because the errors in the remaining classes are not taken into account. Therefore, since our method achieved very high performance for stages N3 and W, while *simultaneously* achieving good performance in the remaining stages, it is preferable to a method that achieves high performance in a stage W vs. N3-only classification task.
